# 805 Case Report: Atrial Perforation in the Setting of Electrical Injury and Subsequent Cardiopulmonary Resuscitation

**DOI:** 10.1093/jbcr/irac012.354

**Published:** 2022-03-23

**Authors:** Kathleen C Gallagher, Robel Beyene, Stephen Gondek, Joshua Smith, Ronnie Mubang

**Affiliations:** Vanderbilt University Medical Center, Nashville, Tennessee; Vanderbilt University Medical Center, Nashville, Tennessee; Vanderbilt University Medical Center, Nashville, Tennessee; Vanderbilt University Medical Center, Franklin, Tennessee; Vanderbilt University Medical Center, Nashville, Tennessee

## Abstract

**Introduction:**

Electrical injuries cause 500 to 1000 deaths per year in the United States and are the fourth leading cause of death in the workplace. Rates and types of cardiac complication secondary to electrical injuries vary widely throughout the literature. Case reports describing cardiac perforation in the setting of electrical injury are exceedingly sparse. To our knowledge, there are no documented cases of cardiac perforation as a direct result of electrical injury.

**Methods:**

This is a case report and review of relevant literature.

**Results:**

Case Description:

We present a case of myocardial perforation in the setting of high voltage electrical injury. An otherwise healthy 46-year old male sustained a 13.8 kV electrical. Resuscitation included bystander cardiopulmonary resuscitation (CPR), defibrillation by emergency medical responders, finger thoracostomies and massive transfusion protocol in the trauma bay, and finally emergent sternotomy and repair of two injuries to the right atrial appendage. Given this patient’s pattern of injury, including a sternal fracture and bilateral upper anterior rib fractures, the etiology of his right atrial perforation is more likely due to mechanical injury from CPR instead of thermal or electrical injury to the myocardium. However, intraoperative findings confound this theory as no defects were identified in the pericardium of the mediastinal pleura during the operation. This would argue against mechanical injury during CPR. However, the presence of a left hemothorax may could indicate that a pericardial injury was missed in the operation. We argue that the most likely cause of injury is mechanical perforation of the pericardium and myocardium during CPR with a possible missed pericardiotomy during surgical exploration. An alternative hypothesis is an isolated electrical injury to the myocardium and left hemothorax from rib fractures without communication between the two cavities. This patient expired on postoperative day two due to anoxic brain injury.

**Conclusions:**

This case highlights myocardial injury as a rare but lethal complication of high voltage electrical injury and serves as a reminder that cardiac perforation and hemodynamic collapse due to hypovolemic shock can compound and complicate the resuscitation and management of a patient with electrical injury. Further, the widely varying data regarding cardiac complications secondary to electrical injury warrants further investigation and large-scale prospective data.

